# Alterations in Circulating miRNAs and Their Potential Role in Aseptic Loosening After Total Hip Replacement: An Observational, Cross-Sectional Study

**DOI:** 10.3390/jpm15110508

**Published:** 2025-10-28

**Authors:** Spyridon Papagiannis, Zinon Kokkalis, George Kyriakopoulos, Antonia Petropoulou, Irini Tatani, Christiana Kotsia, Panagiotis Megas, Constantinos Stathopoulos

**Affiliations:** 1 Department of Orthopedics, School of Medicine, University of Patras, 26504 Patras, Greece; drkokkalis@gmail.com (Z.K.); irinitatani@gmail.com (I.T.); panmegas@gmail.com (P.M.); 2 Department of Biochemistry, School of Medicine, University of Patras, 26504 Patras, Greece; gkyriakop.123@gmail.com (G.K.); toniapetrop99@gmail.com (A.P.); cstath@upatras.gr (C.S.); 3 Department of Radiology, School of Medicine, University of Patras, 26504 Patras, Greece; christiana_kots@hotmail.gr

**Keywords:** miRNAs, aseptic loosening, periprosthetic osteolysis, revision surgery, total hip arthroplasty

## Abstract

**Background/Objectives:** Aseptic loosening (AL) is among the most common causes of late failure following total hip arthroplasty (THA), often necessitating complex revision surgery. Current diagnostic tools, mainly based on clinical and radiological findings, are primarily able to identify advanced changes of periprosthetic osteolysis (PPOL). Therefore, early detection of AL remains a challenge. Circulating microRNAs (miRNAs) have emerged as promising, minimally invasive biomarkers in musculoskeletal disorders. This study investigates the expression of inflammation-related miRNAs let-7i-5p, let-7e-5p, miR-15a-5p, miR-30a-3p and miR-130a-3p in patients with confirmed AL after THA to evaluate their potential role in AL. **Methods:** AL patients undergoing revision were compared with asymptomatic post-THA individuals and controls with degenerative osteoarthritis. Preoperative, peripheral blood samples were collected; total RNA was extracted; and quantitative real-time PCR (qRT-PCR) was performed to quantify miRNA expression. The relative expression of miRNAs was calculated using the 2–ΔΔCt method after proper normalization of Ct values. Statistical analysis assessed differences between groups. **Results:** The under investigation miRNAs exhibited distinct expression patterns. Several targets demonstrated significant downregulation in AL patients, suggesting a potential link to inflammatory and osteolytic pathways like Toll-like receptor 4 (TLR4)–Nuclear Factor kappa-light-chain-enhancer of activated B cells (NF-κB) signaling, NLRP3 inflammasome activation and macrophage polarization. **Conclusions:** The observed alterations in circulating miRNAs support their capability as biomarkers for early detection of AL following THA. Larger cohorts could facilitate translation into routine clinical diagnostics.

## 1. Introduction

Total hip arthroplasty (THA) is the surgical replacement—total or partial—of the hip joint with an artificial implant. Symptomatic osteoarthritis accounts for over 80% of primary THAs, followed by inflammatory arthropathies, femoral head necrosis, fractures, malalignment, and tumor-related bone loss. Candidates are typically aged 60–70 years, with surgery indicated when pain or functional limitation severely impair quality of life [1]. THA is considered one of the most successful and cost-effective interventions in orthopedics, delivering predictable pain relief, functional restoration, and high patient satisfaction. Implant survival averages 15–20 years, with approximately 58% lasting beyond 25 years [2]. In the United States, over 400,000 procedures are performed annually, with demand projected to rise by 129% by 2030 and 284% by 2040 [3,4].

Despite excellent outcomes of primary THA, revision procedures have increased, driven by younger, more active recipients, longer life expectancy, and higher procedural volumes [5]. Revision arthroplasty involves removal, exchange, or addition of prosthetic components and is technically demanding, with longer operative times, higher complication rates, and reduced implant survival [6]. Data from the UK National Joint Registry indicate that approximately 8.95% of hip arthroplasties undergo revision, most commonly for AL(41.4%), periprosthetic fracture (19.7%), infection (9.5%), and instability [7]. Patient factors (age, sex), fixation method, and bearing surface influence revision risk.

AL is the leading cause of late THA failure, arising from biological responses to wear debris and mechanical instability at the bone–implant interface. Periprosthetic osteolysis (PPOL) results from chronic inflammation triggered by implant-derived particulate debris and mechanical stress. Macrophage phagocytosis of wear particles induces pro-inflammatory cytokine release (TNF-α, IL-1β, IL-6), reactive oxygen species, and activation of osteoclast-mediated bone resorption via the RANK/RANKL pathway [8,9,10]. Mechanical factors, such as micromotion and stress shielding, further modulate the inflammatory response. Key cellular contributors include M1 macrophages, osteoclasts, lymphocytes, and neutrophils [11,12,13]. Early detection of AL, prior to radiographic changes, is crucial to prevent implant failure.

MicroRNAs (miRNAs) are small (21–23 nucleotides), single-stranded non-coding RNAs that regulate gene expression post-transcriptionally and have emerged as sensitive, specific, and stable non-invasive biomarkers [14]. They are transcribed by RNA polymerase II, processed by the Drosha–DGCR8 complex, exported to the cytoplasm via Exportin-5, and cleaved by Dicer into ~22-nt duplexes. The guide strand is incorporated into the RNA-induced silencing complex (RISC), mediating target mRNA cleavage or translational repression [15]. Circulating miRNAs are stabilized within exosomes, microvesicles, apoptotic bodies, lipoproteins, or Argonaute proteins, allowing their detection in plasma, serum, urine, saliva, and cerebrospinal fluid and reflecting pathophysiological states [16,17]. Despite their promise, variability in sample type, isolation method, detection platform, and normalization remains a challenge for clinical application [18].

The primary aim of our study was to investigate the relative expression of five inflammation-related miRNAs—let-7i-5p, let-7e-5p, miR-15a-5p, miR-30a-3p, and miR-130a-3p—in patients with AL following primary THA, and to compare these profiles with control patients with degenerative osteoarthritis and asymptomatic THA recipients. A thorough review of recent literature confirmed the involvement of these miRNAs in inflammatory processes contributing to PPOL.

Hsa-let-7i-5p modulates inflammation by targeting TLR4, reducing NF-κB activation and pro-inflammatory cytokine release, while promoting macrophage polarization toward an anti-inflammatory M2 phenotype [19]. Let-7e-5p exhibits context-dependent immunomodulatory effects, suppressing TNF-α, IL-6, TLR4, and NF-κB in some settings, but dysregulation may promote persistent inflammation [20,21]. MiR-15a-5p and miR-30a-3p regulate NF-κB signaling, TLR pathways, and NLRP3 inflammasome activation, with miR-30a-3p also enhancing antioxidant responses via Nrf2; downregulation of these miRNAs may increase macrophage activity and osteoclast-mediated bone resorption [22,23,24,25]. MiR-130a-3p influences TLR4, NF-κB, STAT3, and IRAK1, affecting macrophage polarization and potentially exacerbating peri-implant inflammation [26,27]. Collectively, these miRNAs play central roles in PPOL pathophysiology by modulating inflammatory responses and bone remodeling.

Beyond expression analysis, we assessed the potential of these miRNAs as early biomarkers for AL. Their involvement in key pathways such as TLR4–NF-κB signaling, NLRP3 inflammasome activation, and macrophage polarization highlights their promise as indicators of AL susceptibility. Furthermore, these miRNAs could represent therapeutic targets for pharmacologic or gene-based interventions to control PPOL, reduce inflammation, preserve bone stock, and potentially delay or prevent the need for revision surgery.

## 2. Materials and Methods

### 2.1. Study Design, Study Population, Inclusion and Exclusion Criteria

Three distinct patient cohorts were enrolled in this observational study. Group A included patients over 40 years of age scheduled for primary total THA due to end-stage degenerative osteoarthritis (Tönnis Grade 3) [28]. Group B comprised patients who underwent primary THA for degenerative hip arthritis at least 10 years prior, with no clinical or radiological evidence of PPOL or AL. Group C consisted of patients with clinically and radiologically confirmed AL following primary THA performed at least 10 years prior, who were scheduled for revision arthroplasty.

Inclusion criteria required age >40 years and fulfillment of the respective group-specific clinical and radiological characteristics. Exclusion criteria encompassed any evidence of septic loosening (pre- or intraoperative), prior revision surgery on the affected hip, contralateral total hip replacement or hemiarthroplasty, previous arthroplasty of other major joints (shoulder, knee, elbow, ankle), history of malignancy or immunodeficiency, current chemotherapy or immunosuppressive therapy, autoimmune diseases, severe cognitive impairment or psychiatric disorders, and inability to provide written informed consent.

**Figure 1:**
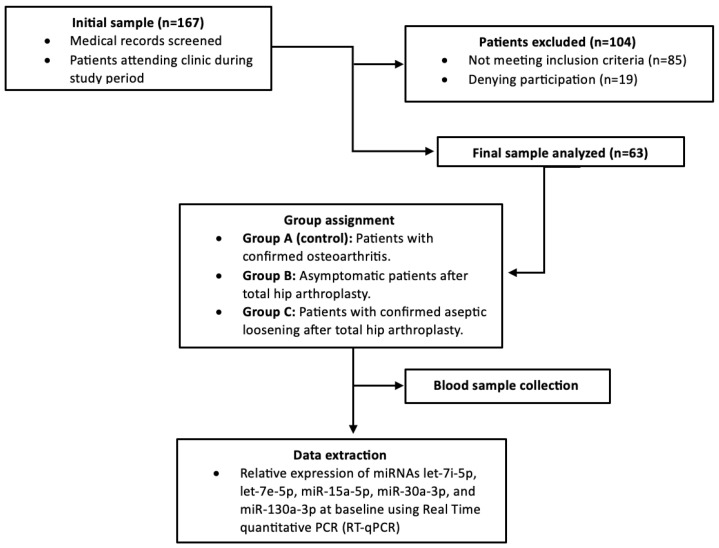
Study flow diagram.

A flow diagram of the study design is presented in Figure 1.

### 2.2. Demographic Characteristics, Statistical Analysis, Sample Size Calculation and Sample Collection

Demographic characteristics, including age, sex, and body mass index (BMI), were recorded, along with clinical parameters such as pain, symptom duration, mechanical symptoms (e.g., instability, locking), and comorbidities. Preoperative medication use, including NSAIDs and anti-osteoporosis agents, did not differ between groups, and no participants were receiving systemic corticosteroids or other immunomodulatory therapies.

Clinical evaluation included assessment of gait, leg length discrepancy, prior surgical incisions, and hip range of motion. Functional status was assessed using the physician-administered Harris Hip Score (HHS) and the patient-reported 36-Item Short Form Health Survey (SF-36), capturing domains of physical function, pain, and overall health perception. Implant-related variables, including bearing surface and fixation method, were documented to ensure comparability between groups.

In Group C, AL was confirmed based on clinical examination and radiographic assessment using anteroposterior pelvis and anteroposterior/cross-table lateral hip views, following established criteria. Implant-related indicators included stem subsidence (>1.5 mm/year), altered stem positioning, cement mantle quality, cup migration, and implant deformation; bone-related features included radiolucent lines, osteolysis, bone remodeling, and heterotopic ossification [29].

Potential infection was systematically excluded in Group C patients through preoperative ESR and CRP measurements (ESR > 30 mm/hr or CRP > 10 mg/L considered concerning) and five intraoperative tissue cultures. As all patients were scheduled for revision arthroplasty, preoperative joint aspiration was not performed.

Finally, a power analysis was conducted to ensure adequate sensitivity for detecting differences in miRNA expression. Continuous miRNA measurements were compared among the three patient groups using one-way ANOVA, followed by Bonferroni post hoc tests for pairwise comparisons. Considering the logical ordering of the groups, secondary analyses were performed by merging adjacent groups (A–B vs. C or A vs. B–C), enabling ROC curve analysis to determine optimal cutoffs and evaluate diagnostic performance. Logistic regression was employed to assess the predictive value of multiple miRNAs for binary outcomes derived from these merged groups.

To account for potential confounders, additional multivariable analyses were performed. Linear regression models were constructed with ΔCt values as the dependent variable, including age, sex, BMI, time since THA, bearing surface, and fixation method as covariates. These adjustments allowed assessment of the independent association between each miRNA and AL. The adjusted analyses confirmed the trends observed in univariate comparisons, demonstrating that the associations of let-7i-5p, miR-30a-3p, and miR-130a-3p with AL remained robust after controlling for these variables.

Sample size calculations were performed using the R ‘pwr’ package, assuming a large effect size to reflect substantial group differences. Based on these calculations, 63 patients (21 per group) provided 80% power at α = 0.05. This approach guided the selection of our study cohort, ensuring sufficient statistical power while considering practical and budgetary constraints. The main demographic and functional characteristics of the study population are presented in Table 1.

Peripheral blood samples were obtained from all participants. Patients in Groups A and C were sampled preoperatively, while synovial membrane specimens were collected intraoperatively. Group B participants provided only blood samples, as they were not undergoing surgery. All samples were collected in the morning after overnight fasting to minimize circadian and dietary influences.

Hemolysis was assessed visually and spectrophotometrically at 414 nm, and any hemolyzed samples were excluded. Blood was collected in serum separator tubes (silica clot activator with polymer gel), allowed to clot for 20–30 min, and then centrifuged at 2000× *g* for 10 min. Serum was aliquoted into RNase-free microcentrifuge tubes, and synovial tissue samples were stored in sterile containers. All samples underwent a single freeze–thaw cycle and were stored at −70 °C until analysis.

### 2.3. MiRNA Isolation from Serum Samples and Synovial Tissues

After initial centrifugation serum samples were recentrifuged at 12,000× *g* for 10 min to remove residual platelets and cellular debris. Total circulating miRNA was then isolated using the NucleoSpin miRNA Plasma Kit (Macherey-Nagel GmbH & Co. KG, Düren, Germany ) according to the manufacturer’s instructions. To minimize RNA degradation, an RNase inhibitor ( RNasin® Plus, Promega, Madison, WI, USA) was added to the lysis buffer at a final concentration of 1 U/μL, following standard recommendations.

RNA yield and purity were quantified spectrophotometrically at 260/280 nm using a CARY 1E UV–Vis spectrophotometer (Varian, Palo Alto, CA, USA), and samples with A260/A280 ratios between 1.8 and 2.1 were considered acceptable. RNA integrity was also evaluated using the Agilent 2100 Bioanalyzer(Agilent Technologies, Santa Clara, CA, USA), and samples displaying RIN values consistent with good-quality RNA (typically 7.0–8.5) were selected for qRT-PCR analysis.

Synovial tissue samples consistently yielded RNA of insufficient quantity and quality, primarily due to high RNase activity, small sample size, and incomplete homogenization. Consequently, these samples were excluded from downstream analyses. Periprosthetic tissues mainly comprise fibrous connective tissue, inflammatory infiltrates, necrotic debris, and variable amounts of mineralized bone. Tissue components were processed following mechanical disruption; mineralized elements underwent EDTA or acid decalcification, whereas formalin-fixed paraffin-embedded (FFPE) samples were deparaffinized prior to extraction. Rapid freezing and RNA-stabilizing agents were employed to preserve RNA integrity [30]. Small RNA enrichment protocols were additionally applied, although partially mineralized or very small tissue fragments remained suboptimal sources.

### 2.4. Reverse Transcription and Quantitative Polymerase Chain Reaction (qPCR) Assay

Equalized RNA inputs were polyadenylated (Poly(A) Tailing Kit, Thermo Fisher Scientific Inc., Santa Clara, CA, USA), purified, and reverse-transcribed using an oligo-dT adapter primer (BioScript™ Reverse Transcriptase). MiRNA expression was quantified by qRT-PCR (KAPA SYBR FAST, MX3000P, Agilent Technologies, Santa Clara, CA, USA) in randomized run order, with inter-plate calibrators to correct for batch effects. MiRNA-specific primers were designed according to miRBase IDs and validated for efficiency (Table 2). All reactions were performed in triplicate, with intra- and inter-assay variability assessed using control samples to ensure accurate and reproducible quantification of circulating miRNA levels.

Ct values were averaged from triplicates and analyzed using the 2−ΔΔCT method, normalized to RNU6 and calibrated to controls (MxPro software, version v4.10d, Agilent Technologies, Santa Clara, CA, USA)). Small RNA controls were additionally employed for miRNA expression normalization; however, no universally validated endogenous reference genes exist for periprosthetic tissue, and commonly used controls (e.g., U6, SNORD44) may be influenced by inflammatory processes [31,32].

### 2.5. Statistical Analysis

Results are presented as relative expression levels normalized to the mean values of the control replicates. Statistical analyses were performed using GraphPad Prism version 8.0.2 (GraphPad Software Inc., San Diego, CA, USA). Comparisons between groups were conducted using unpaired Student’s t-tests, with data reported as mean ± SD from three independent biological replicates. Statistical significance was defined as *p* < 0.05. Additional details are provided in the corresponding figure legends.

## 3. Results

### 3.1. Differential Expression of Let-7i-5p in AL and Post-THA Patients

**Figure 2:**
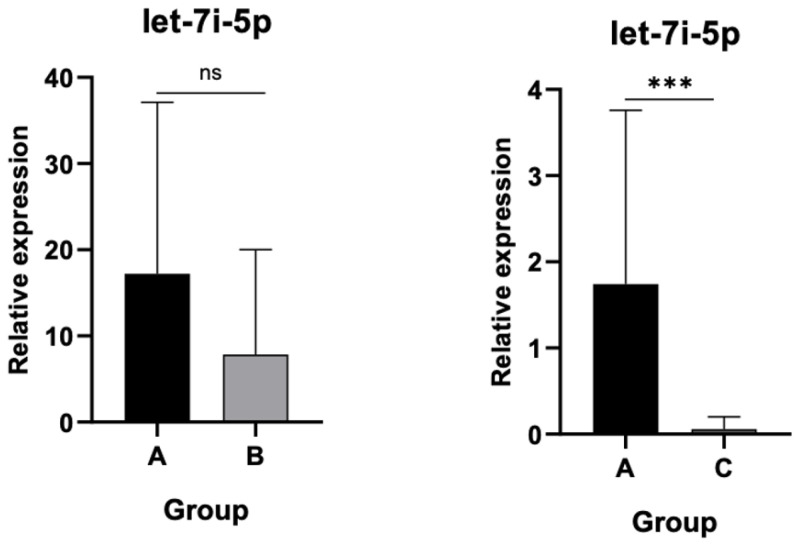
Relative expression of let 7i-5p. Significant downregulation in patients with AL (Group C) compared to osteoarthritis controls (Group A) (*p* < 0.001). ns: not significant, (***): statistically significant, *p* < 0.001

Quantitative analysis revealed that let-7i-5p expression was significantly downregulated in patients with AL (Group C) compared to control patients with osteoarthritis (Group A) (*p* < 0.001), as shown in Figure 2. Although expression in asymptomatic post-THA patients (Group B) was reduced relative to controls, this difference did not reach statistical significance. Moreover, let-7i-5p levels were significantly higher in Group B than in Group C (*p* < 0.01), implicating this miRNA in the pathophysiology of aseptic loosening.

### 3.2. Profound Decrease of Let-7e-5p and miR-15a-5p Levels in Both Asymptomatic Post-THA Recipients and AL Patients

**Figure 3:**
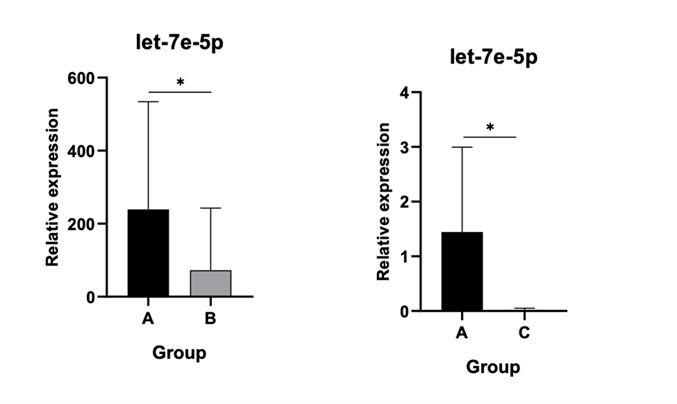
Relative expression of let 7e-5p. No significant differential expression observed between the two patient cohorts. (*): statistical significance, *p* < 0.05

Let-7e-5p demonstrated a consistent and significant decrease in expression in both Group B and Group C relative to controls (*p* < 0.05) as depicted in Figure 3, with no significant differential expression observed between the two patient cohorts.

**Figure 4:**
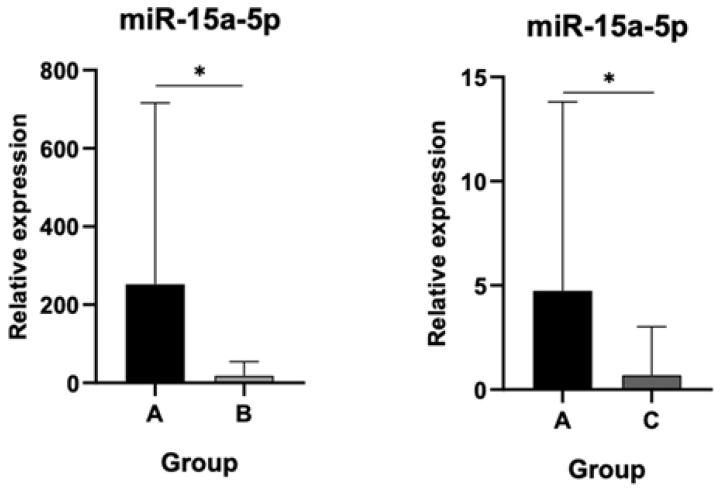
Relative expression of miR-15a-5p. No statistically significant distinction was observed between Groups B and C. (*): statistical significance, *p* < 0.05

The expression of miR-15a-5p was significantly attenuated in both asymptomatic and AL groups compared to controls (*p* < 0.05) as demonstrated in Figure 4, yet no statistically significant distinction was observed between Groups B and C.

### 3.3. Differential Expression Levels of miR-30a-3p and miR-130a-3p Among Cohort Groups

**Figure 5:**
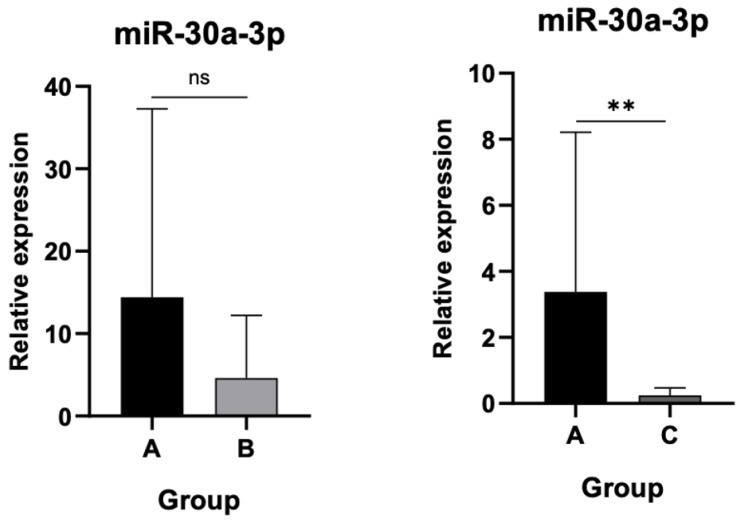
Relative expression of miR-30a-3p. Significant downregulation in Group C compared to controls (*p* < 0.001). ns: not significant, (**): statistically significant, *p* < 0.001

Significant downregulation of miR-30a-3p was detected in Group C compared to controls (*p* < 0.001), whereas the reduction in Group B did not achieve statistical significance as shown in Figure 5.

**Figure 6:**
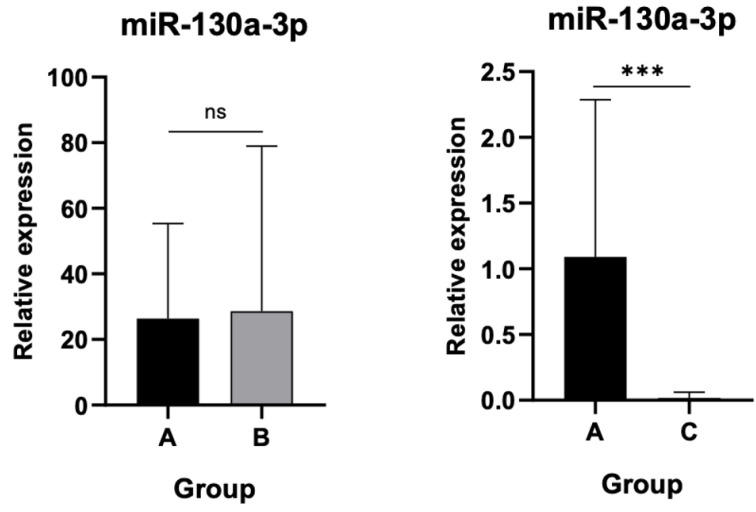
Relative expression of miR-130a-3p. Marked decrease in Group C compared to controls (*p* < 0.001). ns: not significant, (***): statistically significant, *p* < 0.001

Finally, miR-130a-3p expression remained statistically unchanged between asymptomatic patients and controls but was markedly decreased in the AL group compared to controls (*p* < 0.001) as indicated in Figure 6.

### 3.4. MiRNA Expression Profiles Distinguishing AL from Asymptomatic Post-THA Patients

**Figure 7:**
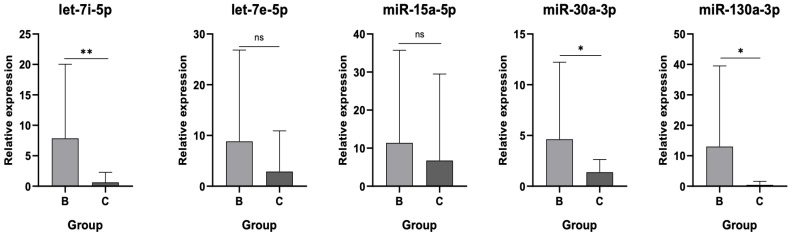
Relative expression of all 5 miRNAs between Groups B and C. Statistically significant downregulation of let-7i-5p, miR-30a-3p and miR-130A-3p is patients with AL. ns: not significant, (*): statistical significance, *p* < 0.05, (**): statistical significance, *p* < 0.01

Comparison of all five selected miRNAs between asymptomatic post-THA patients (Group B) and those with AL (Group C) is summarized in Figure 7. Let-7i-5p expression was significantly higher in Group B than in Group C (*p* < 0.01), indicating pronounced downregulation in AL. Similarly, miR-30a-3p and miR-130a-3p were significantly lower in Group C compared to Group B (*p* < 0.05), reflecting a consistent pattern of reduced expression associated with implant failure. In contrast, let-7e-5p and miR-15a-5p did not differ significantly between groups, although a trend toward lower levels in Group C was observed.

## 4. Discussion

Despite the overall high success of THA, complications such as periprosthetic joint infection, dislocation, venous thromboembolism, and leg length discrepancy may occur [33,34]. Nevertheless, AL remains the leading cause of revision surgery, underscoring the importance of early detection to prevent progressive bone loss and enable timely intervention. Clinical symptoms -including insidious hip pain and functional impairment- are often nonspecific, necessitating comprehensive evaluation. Conventional radiography is typically used for initial assessment, while advanced imaging modalities (CT, MRI, SPECT/CT, PET) can improve diagnostic sensitivity, though radiographic changes often appear at advanced stages. This limitation highlights the need for sensitive and specific biomarkers for early AL detection. Among emerging molecular candidates, circulating microRNAs are particularly promising due to their stability and regulatory roles in inflammation, bone remodeling, and immune signaling [35,36].

In this study, let-7i-5p was significantly downregulated in AL patients compared to both osteoarthritis controls and asymptomatic post-THA individuals. This downregulation may disinhibit TLR4 and NF-κB signaling, increasing pro-inflammatory cytokines such as IL-6 and TNF-α, which promote osteoclast differentiation via the RANK/RANKL/OPG axis. Let-7 family also negatively regulates the NLRP3 inflammasome, limiting IL-1β secretion; its suppression could thus sustain osteoclastic activity and macrophage polarization toward the pro-inflammatory M1 phenotype, perpetuating periprosthetic osteolysis (PPOL) [37]. Although TLR4/NF-κB signaling and cytokine levels were not directly measured, the observed miRNA pattern aligns with these proposed mechanisms, warranting future studies integrating miRNA and protein-level analyses.

Additionally, miR-30a-3p and miR-130a-3p showed stepwise downregulation in AL patients, suggesting roles in PPOL pathogenesis. MiR-30a-3p regulates bone metabolism via RUNX2 and Smad pathways; its suppression impairs osteoblast function, enhances autophagy, and increases NF-κB–mediated inflammation, promoting RANKL expression and osteoclastogenesis [38,39]. MiR-130a-3p modulates M1 macrophage polarization, osteoclast activity through NFATc1, and inflammatory signaling via STAT3, affecting IL-6 and TNF-α production while influencing angiogenesis and tissue remodeling [40]. Though these pathways were not directly measured, integration with observed miRNA expression provides a biologically plausible framework linking miRNA dysregulation, chronic inflammation, and bone resorption in AL. The summarized functions and pathological implications of these miRNAs are presented in Table 3.

On the contrary, miRNAs let-7e-5p and miR-15a-5p were significantly downregulated in both Groups B and C compared to control Group A patients. However, no statistically significant difference in the expression levels of these miRNAs was reached between asymptomatic post-THA individuals and revision arthroplasty candidates due to AL. This pattern likely reflects a generalized molecular response to implantation, rather than a direct association with the molecular pathways of particle-induced osteolysis.

Understanding the distinct pathogenic mechanisms of osteoarthritis (OA) and AL is essential for interpreting molecular biomarkers. OA is driven by mechanical stress, age-related cartilage wear, and metabolic changes, with damage-associated molecular patterns (DAMPs) activating synoviocytes, chondrocytes, and macrophages, leading to low-grade inflammation, cytokine release (IL-1β, TNF-α, IL-6), matrix degradation, and subchondral bone remodeling [41,42]. In contrast, AL results from a foreign body reaction to implant-derived wear particles, activating macrophages via TLR4, triggering NF-κB and NLRP3 inflammasome signaling, and promoting RANKL-mediated osteoclastogenesis and periprosthetic bone loss. While some pathways overlap, OA is characterized by systemic low-grade inflammation and cartilage degeneration, whereas AL involves localized, particle-driven inflammation with prominent bone resorption. Accordingly, circulating miRNA profiles differ between these conditions, with the observed downregulation of let-7i-5p, miR-30a-3p, and miR-130a-3p likely reflecting the unique inflammatory and osteolytic pathways of AL rather than OA.

Beyond their diagnostic potential, let-7i-5p, miR-30a-3p, and miR-130a-3p may serve as therapeutic targets for early intervention in AL. Restoration of their expression via miRNA mimics, nanoparticle-based delivery, or viral vector-mediated gene transfer could provide a localized, targeted approach to mitigate disease progression, given their significant downregulation and roles in key osteolytic and inflammatory pathways. The feasibility of miRNA-based therapies is supported by ongoing clinical studies in oncology (e.g., MRX34, a miR-34a mimic) and infectious diseases (e.g., anti-miR-122 for hepatitis C) [43,44,45,46]. In bone research, preclinical studies demonstrated modulation of osteoclastogenesis via miRNAs such as miR-214 through the PI3K/Akt pathway, highlighting their therapeutic potential [47].

Translation into clinical practice, however, faces challenges including efficient and tissue-specific delivery, minimizing off-target effects, and ensuring sustained and regulated expression. Nonetheless, the miRNAs identified in this study represent promising candidates for dual application—as minimally invasive biomarkers and as therapeutic targets—supporting precision medicine strategies for early intervention and improved long-term outcomes following total hip arthroplasty.

While the observed alterations in circulating miRNA expression provide insights into potential molecular mechanisms and may suggest therapeutic relevance, these interpretations remain speculative. The lack of synovial tissue data and absence of functional assays preclude confirmation of causality or mechanistic involvement. Future research integrating tissue-level expression profiling and in vitro functional validation is warranted to clarify the biological significance of these miRNAs and to evaluate their potential as therapeutic targets in periprosthetic osteolysis.

Despite promising results, several limitations should be acknowledged. First, patients in Groups B and C received prostheses with diverse bearing surfaces (metal-on-metal, metal-on-polyethylene, ceramic-on-ceramic, ceramic-on-polyethylene) and fixation methods (predominantly cementless, with some hybrid), with variable time since THA. Such heterogeneity may influence wear particle generation and inflammatory responses, potentially affecting circulating miRNA profiles [48]. Although implant outcomes depend on overall construct performance, residual confounding could have biased our results. Future larger, multicenter studies could allow stratified analyses to disentangle the effects of bearing surface, fixation type, and postoperative duration.

Second, the relatively small sample size—particularly in the AL group—may limit statistical power and generalizability, reflecting strict inclusion criteria and the limited number of revision hip arthroplasties at our institution. Third, the cross-sectional design with a single postoperative miRNA measurement precludes assessment of temporal expression dynamics. Fourth, circulating miRNA levels are subject to pre-analytical, analytical, and biological variability, including sample handling, detection efficiency, normalization strategy, and patient-related factors, which should be standardized for reproducibility [49]. Finally, this study focused on five preselected inflammation-related miRNAs, potentially overlooking other relevant candidates; unbiased high-throughput profiling may identify additional biomarkers and improve diagnostic precision.

Based on these limitations and preliminary findings, future research should aim to: (1) increase reproducibility and statistical robustness via larger, multicenter cohorts; (2) standardize and stratify implant- and surgery-related variables to isolate disease-specific miRNA signatures; (3) conduct longitudinal sampling alongside radiographic, clinical, and biochemical data to characterize temporal miRNA dynamics; (4) develop multi-marker diagnostic algorithms combining expanded miRNA profiling with established biomarkers; (5) establish standardized pre-analytical and analytical protocols; (6) explore broader applicability across other joint replacements and implant-related complications such as periprosthetic joint infection; and (7) evaluate clinical feasibility, including cost-effectiveness, turnaround times, and integration into routine orthopedic follow-up.

## 5. Conclusions

AL remains the leading cause of long-term failure after total hip arthroplasty, while current diagnostic strategies depend largely on imaging modalities that typically identify implant failure only after significant bone loss has occurred. In this context, our study highlights the potential of circulating microRNAs—specifically let-7i-5p, let-7e-5p, miR-15a-5p, miR-30a-3p, and miR-130a-3p—as early, non-invasive biomarkers that may capture the molecular mechanisms driving periprosthetic osteolysis. Notably, the downregulation of let-7i-5p, miR-30a-3p, and miR-130a-3p in patients with aseptic loosening suggests an active involvement in inflammatory signaling, osteoclast activity, and macrophage polarization.

By applying strict inclusion criteria and well-defined comparison groups, this work addresses several methodological challenges commonly encountered in circulating miRNA research and provides a foundation for future longitudinal and functional studies. These results support the emerging paradigm that miRNAs can serve a dual role—not only as minimally invasive diagnostic indicators but also as potential therapeutic targets. While translation into clinical practice will require validation in larger, multicenter cohorts and strategies to overcome delivery and reproducibility barriers, our findings offer a promising step toward precision monitoring and novel treatment approaches in orthopedic implant complications.

## Figures and Tables

**Table 1:** Baseline demographic and functional characteristics of our study population.

Variable	OA Group (*n* = 21)	THA Group (*n* = 21)	REV Group (*n* = 21)
Gender (F/M)	12/9	15/6	15/6
Age (years)	64.3 (58–76)	76.9 (69–85)	75.4 (63–81)
BMI (kg/m^2^)	26.8 (23.2–34.7)	24.6 (22.1–29.3)	26.9 (23.6–32.8)
Time since primary THA (years)	—	13.8 (12–16)	14.7 (11–20)
Bearing surface	—	MoM: 13, CoC: 5, MoP: 3	MoP: 11, CoP: 6, MoM: 4
Type of fixation	-	Cementless: 16, Hybrid (cemented stem: 5)	Cementless:18, Hybrid (cemented stem: 3)
Preoperative HHS	63.9 (52.8–77.6)	82.3 (73.4–89.2)	53.5 (41.4–63.9)
Preoperative SF-36—General health perception (%)	58.6 (49–71)	80.8 (71–86)	50.2 (39–62)
Preoperative SF-36—Physical functioning (%)	61.6 (52–74)	82.6 (76–88)	55.7 (49–70)

Values are presented as mean (range). MoM: metal-on-metal, CoC: ceramic-on-ceramic, MoP: metal-on-polyethylene, CoP: ceramic-on-polyethylene; HHS: Harris Hip Score.

**Table 2:** List of primers and DNA oligos used in the present study.

Primer Name	Sequence (5′ → 3′)
Oligo-dT adapter	GCGAGCACAGAATTAATACGACTCATATAGGTTTTTTTTTTTTVN
Outer Primer	GCGAGCACAGAATTAATACGACT
RNU6	GCTCGCTTCGGCAGCACATA
let-7i-5p	TGAGGTAGTAGTTTGTGCTGTT
let-7e-5p	TGAGGTAGGAGGTTGTATAGTT
miR-15a-5p	TAGCAGCACATAATGGTTTGTGAA
miR-30a-3p	CTTTCAGTCGGATGTTTGCAGC
miR-130a-3p	CAGTGCAATGTTAAAAGGGCAT

**Table 3:** Functional roles of downregulated miRNAs in AL.

miRNA	Normal Function	Effect of Downregulation in AL
let-7i-5p	Suppresses TLR4, NF-κB, NLRP3 → restrains inflammation	Enhanced TLR4/NF-κB signaling → sustained macrophage activation and osteolysis
miR-30a-3p	Inhibits autophagy & NF-κB, modulates osteoblast/osteoclast balance	Increased inflammation, impaired osteoblast function → net bone loss
miR-130a-3p	Regulates macrophage polarization, suppresses osteoclastogenesis	Shift to M1 phenotype, increased NFATc1 activity → chronic inflammation, bone resorption

## Data Availability

The data presented in this study are available on request from the corresponding author due to privacy reasons.

## Abbreviations

The following abbreviations are used in this manuscript:

Abbreviation	Full Form
AL	Aseptic Loosening
CT	Computed Tomography
DAMPs	Danger-Associated Molecular Patterns
ECmiRNAs	Extracellular Circulating microRNAs
IL-1β	Interleukin 1 beta
IL-6	Interleukin 6
LLD	Leg Length Discrepancy
miRNAs	microRNAs
MRI	Magnetic Resonance Imaging
NF-κB	Nuclear Factor kappa-light-chain-enhancer of activated B cells
OA	Osteoarthritis
OPG	Osteoprotegerin
PAMPs	Pathogen-Associated Molecular Patterns
PET	Positron Emission Tomography
PI3K/Akt	Phosphoinositide 3-kinase/Protein Kinase B
PPOL	Periprosthetic Osteolysis
qRT-PCR	Quantitative Real-Time Polymerase Chain Reaction
RANK	Receptor Activator of Nuclear Factor Kappa-B
RANKL	Receptor Activator of Nuclear Factor Kappa-B Ligand
RISC	RNA-Induced Silencing Complex
RUNX2	Runt-related transcription factor 2
SPECT/CT	Single-Photon Emission Computed Tomography/Computed Tomography
THA	Total Hip Arthroplasty
TLR4	Toll-Like Receptor 4
TNF-α	Tumor Necrosis Factor alpha
VTE	Venous Thromboembolism
